# AI-Driven Insight into Polycarbonate Synthesis from CO_2_: Database Construction and Beyond

**DOI:** 10.3390/polym16202936

**Published:** 2024-10-19

**Authors:** Aritz D. Martinez, Adriana Navajas-Guerrero, Harbil Bediaga-Bañeres, Julia Sánchez-Bodón, Pablo Ortiz, Jose Luis Vilas-Vilela, Isabel Moreno-Benitez, Sergio Gil-Lopez

**Affiliations:** 1 TECNALIA, Basque Research & Technology Alliance (BRTA), Technological Park of Bizkaia, 48160 Derio, Spain; aritz.martinez@tecnalia.com (A.D.M.); adriana.navajas@tecnalia.com (A.N.-G.); pablo.ortiz@tecnalia.com (P.O.); sergio.gil@tecnalia.com (S.G.-L.); 2 Grupo de Química Macromolecular (LABQUIMAC), Departamento de Química Física, Facultad de Ciencia y Tecnología, Universidad del País Vasco UPV/EHU, 48940 Leioa, Spain; harbil.bediaga@ehu.eus (H.B.-B.); julia.sanchez@ehu.eus (J.S.-B.); joseluis.vilas@ehu.eus (J.L.V.-V.); 3 BCMaterials, Basque Center for Materials, Applications and Nanostructures, UPV/EHU Science Park, 48940 Leioa, Spain; 4 Grupo de Química Macromolecular (LABQUIMAC), Departamento de Química Orgánica e Inorgánica, Facultad de Ciencia y Tecnología, Universidad del País Vasco UPV/EHU, 48940 Leioa, Spain

**Keywords:** material science, polymers, copolymerization, artificial intelligence, database, dimensionality problem, machine learning

## Abstract

Recent advancements in materials science have garnered significant attention within the research community. Over the past decade, substantial efforts have been directed towards the exploration of innovative methodologies for developing new materials. These efforts encompass enhancements to existing products or processes and the design of novel materials. Of particular significance is the synthesis of specific polymers through the copolymerization of epoxides with CO_2_. However, several uncertainties emerge in this chemical process, including challenges associated with successful polymerization and the properties of the resulting materials. These uncertainties render the design of new polymers a trial-and-error endeavor, often resulting in failed outcomes that entail significant financial, human resource, and time investments due to unsuccessful experimentation. Artificial Intelligence (AI) emerges as a promising technology to mitigate these drawbacks during the experimental phase. Nonetheless, the availability of high-quality data remains crucial, posing particular challenges in the context of polymeric materials, mainly because of the stochastic nature of polymers, which impedes their homogeneous representation, and the variation in their properties based on their processing. In this study, the first dataset linking the structure of the epoxy comonomer, the catalyst employed, and the experimental conditions of polymerization to the reaction’s success is described. A novel analytical pipeline based on ML to effectively exploit the constructed database is introduced. The initial results underscore the importance of addressing the dimensionality problem. The outcomes derived from the proposed analytical pipeline, which infer the molecular weight, polydispersity index, and conversion rate, demonstrate promising adjustment values for all target parameters. The best results are measured in terms of the (Determination Coefficient) $R^{2}$ between real and predicted values for all three target magnitudes. The best proposed solution provides a $R^{2}$ equal to 0.79, 0.86, and 0.93 for the molecular weight, polydispersity index, and conversion rate, respectively. The proposed analytical pipeline is automatized (including AutoML techniques for ML models hyperparameter tuning), allowing easy scalability as the database grows, laying the foundation for future research.

## 1. Introduction

The continuous growth in anthropogenic CO_2_ emissions, in addition to the clear evidence of their participation in the greenhouse effect, and, thus, in climate change [1,2], have been the driving forces for searching for new alternatives to fix, store, and use them. Fortunately, this gas has a double face because it can be considered an excellent, abundant, and non-toxic one-carbon raw material in organic synthesis and, specifically, in the preparation of certain polymers [3].

Therefore, the use of CO_2_ in the production of these materials is important not only for their high value but also for environmental aspects since emissions can be reduced to a certain extent. However, it must be underlined that the use of CO_2_ in any chemical process, by itself, is not capable of effecting a great impact on the decrease in CO_2_ concentrations in the atmosphere [4].

**Figure 1:**
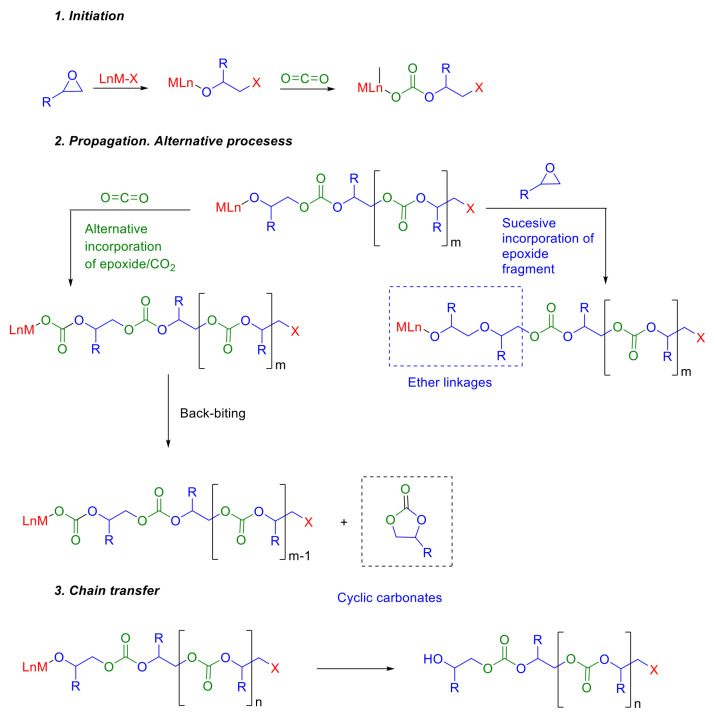
Mechanistic proposal and alternative processes in the synthesis of polycarbonates from epoxides and CO_2_.

The alternating copolymerization of epoxides with CO_2_ in the presence of zinc or cobalt-based catalyst systems, first described by Inoue in 1969 [5], has been considered one of the most promising alternatives for the utilization of CO_2_ in the synthesis of polymers. Three steps including chain initiation, propagation, and chain transfer are involved in this polymerization process (Figure 1). In addition to the desired polycarbonate, the kinetically favored product, two secondary products can be obtained in this method. On the one hand, we have ether linkages, randomly distributed within the polymer backbone due to consecutive ring openings of epoxide without the incorporation of CO_2_. On the other hand, the backbiting process can lead to the formation of cyclic carbonates [3,6]. In this context, the catalyst plays a critical role in determining not only the product distribution but also the molecular weight of the resulting copolymer and, consequently, its properties. In recent years, a variety of catalytic systems have been studied to overcome the inherent thermal stability of CO_2_ [7], providing polycarbonates in good yields [8,9,10,11,12].

The two epoxides used as the benchmark in this copolymerization reaction are propylene oxide (PO) and cyclohexane oxide (CHO) [4]. In this context, it is important to take into account two items. First, the so obtained aliphatic polycarbonates typically display less suitable mechanical and thermal properties compared to aromatic polycarbonates obtained from bisphenol A and phosgene [13]. And, on the other hand, although some advances have been made in the development of bio-based routes to produce these raw materials, currently, epoxides are mainly obtained from petrochemical feedstock, decreasing the greenness of the processes. In consequence, in order to extend the scope of this promising methodology, two topics are imperative including the synthesis of polycarbonates with improved properties and performance results, and the incorporation of bio-based monomers that replace fossil derivatives without detriment to the final polymer properties [14]. Conventionally, the design of new polymers has been a trial and error process. In fact, generally, although new monomers are proposed based on extensive domain knowledge, until the new polymer is synthesized and properly characterized, the success of the polymerization and the properties of the new material are not known. Unfortunately, many times, the proposed reaction does not take place successfully, or, the synthesized materials do not meet the required characteristics for the applications for which they have been conceived. Consequently, significant amounts of funds, manpower and time are required in the process. The absolute revolution that Artificial Intelligence (AI) has caused in daily life, industry and academia is undeniable. Machine Learning (ML), a subfield of AI, deals with analyzing large amounts of data using different algorithms to make predictions [15]. Therefore, the convergence of AI and materials science represents a paradigm change in the design and development of new high-performance polymers. In fact, the application of ML techniques to accelerate the discovery and development of new materials is becoming routine [16,17,18,19]. In this sense, the database construction represents one of the most determining factors in the success of predictive models. In fact, in the application of AI in the design of new materials, one of the greatest difficulties is to achieve a coherent and homogeneous database not only in terms of quality but also in quantity. This is especially difficult in the case of polymeric materials for different reasons. First, a homogeneous representation of polymers is hindered by their stochastic structure. Moreover, their properties can drastically depend on their processing history. Consequently, the available data, still very scarce and disperse, are not given in a common and standardized format. In recent years, progress has been made in this regard. Thus, various accessible databases relate the properties of polymers to their structure. For example, PoLyInfo [20] is one of the biggest databases with a huge amount of data, of which more than 40% are thermal properties of 10 different types of compounds. In recent years, the inference of molecular weight distribution or polymerization conversion using AI techniques has been described. However, in all these previous works, these key properties were predicted based on experimental variables such as polymerization time, temperature or the concentration of the initiator, using a certain monomer in a specific reaction such as the free radical polymerization of methyl methacrylate [21], or the thermal polymerization of styrene [22] or butyl acrylate [23]. However, to the best of our knowledge, to date, no database that relates the structure of the starting monomer to the success of the polymerization reaction has been described.

**Figure 2:**
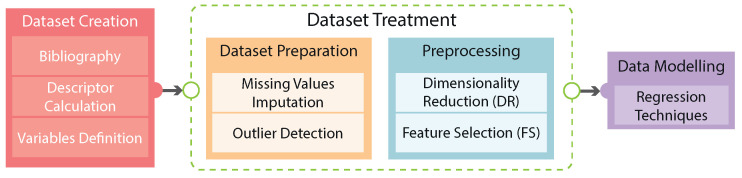
Steps involved in this work.

In this paper, the construction of a database that relates the structure of the epoxy comonomer, the structure of the employed catalyst, and the experimental conditions of the polymerization with the success of the reaction in terms of the molecular weight of the obtained polycarbonate will be described. Subsequently, the methodology applied to infer target variables is proposed. Finally, the manuscript delves into the discussion of the results, where a comprehensive analysis of the outcomes is conducted, leading to the drawing of pertinent conclusions. The flowchart of this work is illustrated in Figure 2.

## 2. Dataset

### 2.1. Description

Due to the synthetic importance of this process, its environmental interest and the potential applications of the obtained polymers, there are many research works found in the literature related to its study. However, the data provided in each of them are very diverse. To create the database, around 15 articles were consulted, in which the copolymerization of an epoxy monomer with CO_2_ in the presence of a catalyst was described [24,25,26,27,28,29,30,31,32,33,34,35,36,37,38]. First, the experimental polymerization conditions, such as pressure, temperature, reaction time, and catalyst percentage were compiled and, obviously, homogenized to the same units. Additionally, the values of *Mn* and its *Mw/Mn* ratio, which provides information on the distribution of molecular weights in the polymer sample, were compiled. Although this first task could be considered tedious, it was relatively easy; each property corresponds to a bibliographic value. On the other hand, once the structures of the monomers and the catalysts used in the available bibliographic sources were collected, the featurization was necessary, that is, the data had to be transformed into a machine-readable format that, if required, could be understood by typical ML algorithms. In this sense, the machine- and human-readable code SMILES [39] (Simplified Molecular Input Line Entry System) was employed to represent the chemical structures of both the monomeric compound and metallic catalyst. Subsequently, the descriptors, or features that represent the molecular characteristics, were obtained, employing the freely available Mordred Python Library [40]. Initially, the 1826 calculated descriptors were included in the database for the epoxy monomer. In contrast, based on the work of Mendes dos Santos [41], only 45 descriptors for each catalyst were calculated. In fact, in this previous work, an analogous set of descriptors, calculated in this case with PaDEL-Descriptor software (http://www.yapcwsoft.com/dd/padeldescriptor/, accessed on 11 September 2024), was selected by the stepwise algorithm because of their great potential for predicting catalytic activity in the carbonation polymerization. Next, those descriptors, both for the monomer and the catalyst, with a value of 0 in all samples, or with a variance less than 0.05, were eliminated.

### 2.2. Data Exploration

In this study, the first analytical pipeline to exploit the previously defined database is described. As said, this is the first database which tries to relate the structure of the monomer to the success of the polymerization reaction. Therefore, three different sources of data are included: (i) structures of the monomers and the catalysts; (ii) experimental polymerization conditions; and (iii) information on the distribution of molecular weights in the resultant polymer. The main objective of the database is to be exploited by ML algorithms to infer the causal relationship between the so-called descriptors (first and second point) and the defined target, i.e., the third referred point.

To understand the scope of the solution, it is important to consider the dimensionality of the dataset (D); it consists of n=201 samples (i.e., different copolymerization of an epoxy monomer with CO_2_ in the presence of a catalyst reactions) and m=1177 features (i.e., descriptors). At this stage, it is important to remark that from those 1177 descriptors, only 3 of them are categorical. This point could affect the learning process of the proposed ML methodologies.

In the data exploration process, a key point to be analyzed is the presence of missing values. In the first exploration, the dataset showed to have a high percentage of missing values, with some descriptors having more than 70% of their data missing. This is a common issue in real-world datasets, and it is important to address it before training a ML model with steps that will be addressed in the next sections. Due to the lack of dimensionality (more descriptors than samples) in the constructed database, the creation of a good missing value imputation methodology will be crucial. Related to the target, the dependent variables are also real values, namely, the molecular weight Mn (kg · mol)^−1^ and the polydispersity index.

**Figure 3:**
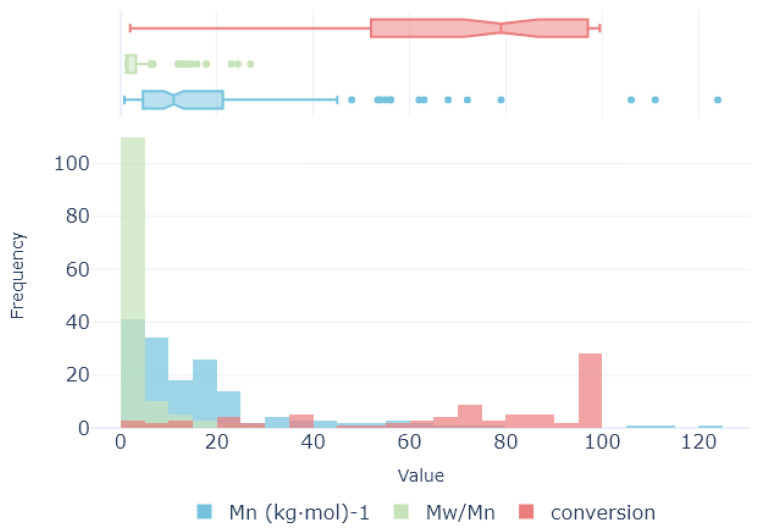
Value distribution for target variables (molecular weight, polydispersity index, and conversion rate).

All target magnitudes are analyzed. The distribution of variables provides valuable insights into their underlying nature. Notably, the variables Mn (kg·mol)^−1^ and $\frac{Mw}{Mn}$ exhibit a considerable number of outlier values (see Figure 3), with the former displaying a particularly pronounced presence. These outliers have the potential to detrimentally impact the performance of certain ML algorithms. On the other hand, the conversion rate shows more coherent distribution with a lack of a significant number of outliers. Thus, the scarcity of samples coupled with the presence of outliers poses a significant challenge for the preprocessing stage. For each target variable, their outliers are detected using 1.5 times their interquartile distance. Then, samples detected as outliers are removed from the training set for each target variable, resulting in a set of 182 samples for Mn (kg·mol)^−1^, 177 for $\frac{Mw}{Mn}$, and 187 for conversion.

Summarizing data exploration, the following can be concluded:

- There is an unfavorable sample-to-descriptor ratio (i.e., massive amount of features (1177) and very few samples (201).
- There is a great amount of missing values (≈12% of the dataset).

The proposed analytical pipeline will focus its endeavors on effectively addressing these two pivotal challenges. The selection of suitable ML algorithms precludes the application of deep learning methodologies, due to insufficient sample size. Consequently, the focus is directed towards traditional ML regression approaches with special emphasis on a new hierarchical missing value imputation algorithm. This latest mentioned algorithm is specially designed to avoid propagating imputation errors to the inferred relationship between descriptor and target magnitudes.

## 3. Proposed Methodology

**Figure 4:**
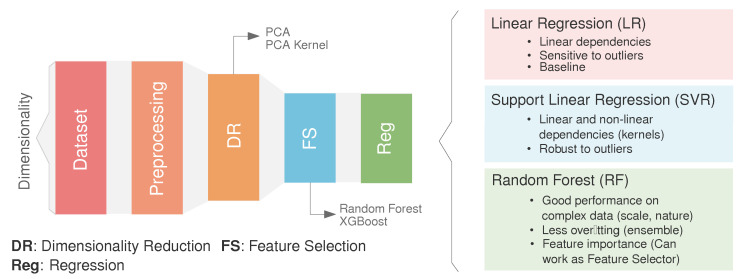
Overview of the technique. The height of the box represent the dimensionality (number of features) at each step (preprocessing, dimensionality reduction, feature selection, and regression). Regression methods considered in this study (right): a. Linear Regression (LR), b. Support Vector Regression (SVR) and c. Random Forest (RF).

In this section, the ML-based methodology is outlined. As illustrated in Figure 4, the methodology comprises four primary stages: (1) preprocessing, involving the preparation and cleaning of the initial dataset, (2) dimensionality reduction (DR), which reduces the dimensions of the initial datasets while considering the intrinsic relationship between the features, (3) feature selection (FS), where a secondary reduction stage is developed by selecting the most representative features in the inference process, and (4) regression optimization, where the target features are predicted using three well-known algorithms. Through the following subsections, each of the stages is described in depth.

### 3.1. Data Preprocessing

For ensuring the quality of the data in the learning process, data preprocessing techniques are included in the analytical pipeline. Data preprocessing comprises a set of methodical procedures executed prior to the application of any ML algorithm, aimed at refining and optimizing raw data for subsequent analysis. These tasks encompass various operations including but not limited to data cleaning, handling missing data, or data normalization.

**Figure 5:**
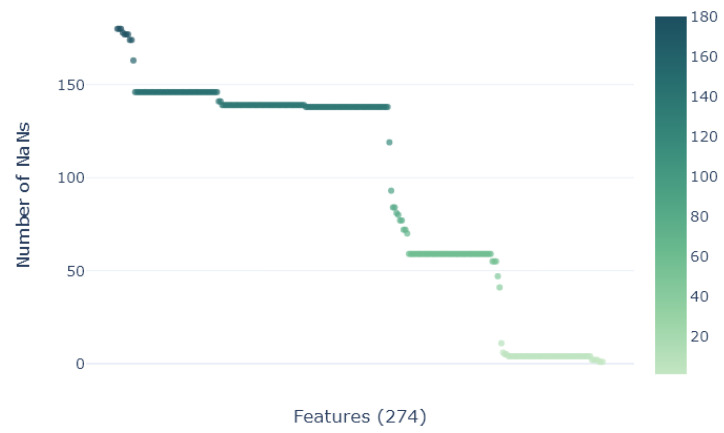
Number of NaNs for each of the 274 descriptors/features shown to contain missing values.

As illustrated in Figure 5, the database reveals a significant presence of missing values across 274 descriptors out of a total of 1177. Completely removing samples with missing values, the dimensionality of the database would be reduced to 12 complete samples, which would exacerbate the dimensionality problem. A new customized iterative methodology (Algorithm 1) characterized by minimal error propagation is employed to effectively compute the remaining missing values.
**Algorithm 1:** Missing value imputation algorithm.
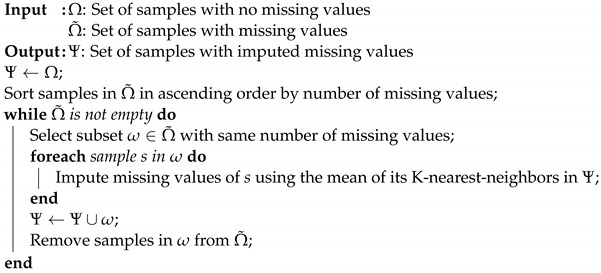


The proposed hierarchical missing value imputation method described in Algorithm 1 starts by initializing the imputed sample set Ψ with those samples devoid of any missing values, carrying out this process hierarchically from those with the fewest missing descriptors to the most. The proximity to the K-Nearest-Neighbors (KNN) [42] is assessed solely based on samples exhibiting fewer missing values relative to the given point. KNN is based on the Euclidean distance between samples. Given a NaN in Ψ, all other descriptors with no-NaNs are used to find its KNN. From ω (i.e., k-neighbors), their mean value of the descriptor to be imputed is used to fill the NaN. Iteratively, the values of the samples with fewer missing values ω are imputed and inserted to Ψ, guaranteeing at each step minimal error propagation derived from imputation. Due to the nature of the KNN, descriptors exhibiting more than 70% missing values are systematically eliminated, as it is no guarantee that they will provide a realistic data distribution on imputation and they will introduce noise to nearly every sample. In this process, 59 descriptors are removed, keeping 1118 out of the initial 1177 descriptors for the missing value imputation phase.

Through this methodology, the integrity of the database’s characteristics is upheld while ensuring a substantial proportion of samples remain available for the training phase.

### 3.2. Dimensionality Reduction

Once the database is curated, dimensionality reduction techniques are essential in ML inference for several reasons. Firstly, as the database has very high dimensions, with many descriptors in comparison with samples, learning inference can be computationally expensive and can lead to overfitting. By reducing the number of dimensions, the data can be simplified without losing important information, making them easier and faster to analyze and model. Additionally, they can improve the performance of ML algorithms by reducing noise, multi-collinearity, and the curse of dimensionality.

In this study, for enhancing the efficacy of learning procedures, two well-known dimensionality reduction techniques are applied, i.e., Principal Component Analysis (PCA) as the linear approach and PCA-Kernel as the non-linear approach.

- PCA is one of the most commonly used dimensionality reduction techniques. It works by finding the orthogonal axes (principal components) along which the variance of the data is maximized [43]. These principal components capture the directions of maximum variance (related to learning capabilities) in the data. PCA then projects the original data onto these principal components, effectively reducing the dimensionality while preserving the most significant variation in the data. Some of its potential qualities relates to (1) its linearity, as PCA works well when the relationship between features are linear; (2) its computational efficiency and wide applicability; and (3) its assumption that the data are centered around the origin.
- PCA-Kernel is an extension of PCA that allows non-linear dimensionality reduction [44]. It applies the kernel trick, similar to Support Vector Machines [45], to project the data into a higher-dimensional space, where they becomes linearly separable. PCA (as explained above) is then performed in this higher-dimensional space, followed by projecting the data back to the original space.

In summary, while PCA is suitable for linear dimensionality reduction, PCA-Kernel is preferred for handling non-linear relationships in the data, albeit at a higher computational cost. Each technique is applied based on the specific characteristics and objectives of the dataset at hand.

### 3.3. Feature Selection

Feature Selection and dimensionality reduction are both techniques used to simplify and improve ML models, but they operate differently and serve distinct purposes. While, as said, the primary goal of dimensionality reduction is to reduce the number of descriptors (or dimensions) in the database by creating new, uncorrelated variables, feature selection involves isolating the most consistent, non-redundant, and relevant descriptors for utilization in ML model construction.

In this study, two well-known methods for feature selection are implemented. Both methods are based on ensemble learning, which utilizes statistical theory to conjugate multiple individual learners:

- Random Forest assigns feature importance scores based on how much each feature decreases the impurity in the individual trees of the ensemble forest. Features with higher importance scores are considered more significant and are thus retained for subsequent modeling tasks [46].
- XGBoost, an implementation of gradient boosting, works by sequentially adding decision trees to an ensemble model, with each subsequent tree trained (by employing gradient descent techniques) to correct the errors of the previous ones [47].

### 3.4. Regression Techniques

Regression analysis is a fundamental concept in statistics and ML, utilized to understand the causal relationship between one or more independent descriptors and a dependent target variable (in the present study, the molecular weight (Mn (kg·mol)^−1^), the polydispersity index ($\frac{Mw}{Mn}$), and the conversion rate (conversion)). Its primary objective is to predict the value of the dependent variable based on the values of the given set of descriptors. This can help domain experts select experiments with a higher likelihood of success, avoiding the trial and error approximation.

Due to the previously mentioned characteristics of the newly constructed database (i.e., dimensionality problem), in this study, three of the most popular and well-known shallow learning regression techniques are selected, being deep learning counterparts that are clearly counterproductive due to data scarcity. Choosing the appropriate regression method depends on the nature of the problem, the characteristics of the dataset, and the desired level of interpretability versus prediction accuracy. The selection of the algorithms is made based on considering an increase in their complexity, from linear methods, Support Vector Machines, to ensemble methods, which could help the reader to understand the underlying relationship between descriptors and targets:

- Linear Regression (LR) [43] assumes a linear relationship between the descriptors and target variables. It aims to fit a straight line to the data that best minimize the sum of squared residuals.
- Support Vector Regression (SVR) [48] aims to use kernel functions to transfer the original descriptor space to a higher dimensional one, which enables samples to exhibit linear separability. It employs the Maximum Margin criterion.
- Random Forest (RF) [46] is based on ensemble learning. By combining the predictions of multiple decision trees, RF reduces overfitting and generalizes well to unseen data, improving predictive performance and robustness. It is less sensitive to noisy data and outliers compared to individual decision trees.

## 4. Experiments Results

### 4.1. Experiments Description

**Figure 6:**
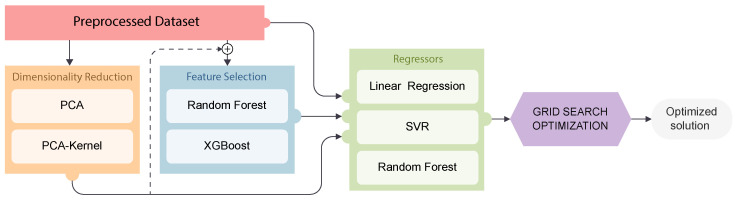
Enhanced methodology: Preprocessed data can be directly fed into the regression models, or they can undergo a combination of Dimensionality Reduction (orange box) and Feature Selection (blue box) processes. For each combination, a Grid Search optimization algorithm is applied to select the best parameter combination for each regressor.

In summary, the proposed analytical pipeline shown in Figure 6 encompasses four key stages, preprocessing (outlier identification and NaNs filling), dimensionality reduction, feature selection, and predictive regression modeling, each outlined in the preceding section. To maximize the effectiveness of the proposed regression schemes, it is important to select the best-suited hyperparameters of each model. To achieve this, Grid Search optimization enables each model to adjust their inference capabilities to the specific characteristics of the built database. The experimental parameters in this study are summarized in Table 1.

As depicted in Figure 6, the proposed analytical pipeline consists of three main stages. Initially, outlier elimination and zero filling are performed. Subsequently, depending on the nature of the relationship between the descriptors and targets, not all stages may be necessary. A benchmark study is presented; different combinations are then systematically evaluated against the three proposed regression models. The evaluated combinations include (from Figure 6); (i) direct evaluation of the preprocessed dataset without dimensionality reduction nor feature selection (red), (ii) dimensionality reduction using PCA or PCA-Kernel (red ⊕ orange), (iii) feature selection using Random Forest or XGBoost (red ⊕ blue), and (iv) dimensionality reduction combined with feature selection (red ⊕ orange ⊕ blue). All defined combinations, as mentioned, have optimization methodologiesto adjust their hyperparameters, and thus learning capabilities, to the characteristics of the defined analytical pipeline. This proposed analytical pipeline enables each model to achieve optimal results when trying to predict the molecular weight Mn (kg·mol)^−1^ and $\frac{Mw}{Mn}$, and the conversion rate (conversion) in a copolymerization of an epoxy monomer with CO_2_ in the presence of a catalyst, departing from a newly defined database.

Generally, the performance of an ML pipeline is measured dividing the database into two different groups, the training and the validation datasets. The training data are used to select the most appropriate hyperparameters of the models and the validation data are used to measure their performance while generalizing their behavior in an unseen dataset. Selecting an appropriate training schema becomes a matter of the utmost importance, especially in the context of a dataset with limited samples like the one at hand. This allows the scheme to balance the variance and bias error’s contributions. With this consideration in mind, the chosen validation technique is Leave-One-Out Cross-Validation (LOOCV). This method proves particularly beneficial when working with a restricted amount of training data because the size of the training data is maximized, ensuring the biggest learning capacities. In LOOCV, only one sample of the dataset is used as the validation set, while the rest of the data are used as the training set. This process is iteratively repeated until all samples are tested. This procedure is repeated for each data point within the database, leading to a series of training and evaluation rounds equivalent to the total number of data points. By systematically leaving out one data point at a time, LOOCV provides a comprehensive assessment of the model’s performance. This methodology is applied individually and independently for each target variable, namely, i.e., Molecular weight Mn (kg·mol)^−1^, polydispersity index ($\frac{Mw}{Mn}$), and conversion rate. To assess the model’s quality, in this study, the Determination Coefficient $R^{2}\in R\left(-\infty ,1\right)$ is employed. This metric represents the proportion of the variation in the dependent variables Mn (kg·mol)^−1^, $\frac{Mw}{Mn}$ and conversion that are predictable from the independent variables (descriptors). Notably, a value closer to 1.0 denotes heightened predictability and, thus, superior model quality. On the other hand, values under 0 denote a lack of predictability, indicating that the model fails to capture the variation in the target (dependent) variables based on the provided descriptors.

### 4.2. Results

Delving into the statistical analysis of the results, the performance of the defined analytical pipelines is shown in Table 2 for each of the defined target magnitudes (different columns), i.e., (Mn (kg·mol)^−1^, $\frac{Mw}{Mn}$ and conversion rate). Every row within the table delineates the diverse combinations of models which are included in each analytical pipeline employed for preprocessing and transforming the data, i.e., raw dataset. The RAW preprocessing stage is universally applied across all combinations but omitted for brevity. Subsequently, the dimensionality reduction phase is represented, commencing with the PCA identifier, followed by any kernel methods poly, rbf, if applicable, and ending with the methodology employed for determining the number of principal components, whether by sample size “sam” or by explained variance “var”. Lastly, if feature selection is conducted, it is distinctly denoted by their respective types, “RF” for Random Forest and “XGB” for XGBoost. These combinations are denoted by a concatenation of their respective identifiers. The mean error values obtained from the LOOCV validation procedure are shown for 15 different experimental runs (SEEDS: 645, 4578, 72, 2365, 90345, 24, 1859, 1334, 2078, 2446, 7409, 6995, 2041, 449, and 9475), i.e., for each combination of data and regressor per target variable.

As it can be clearly shown in Table 2, the analytical pipelines which obtain superior performance are those ones which include RF as the model regressor, obtaining the highest $R^{2}$ values, indicating the best explanatory capacity. Its counterpart, Linear Regression (LR), is the worst model in a generalized way, although the rate conversion is the target magnitude which exhibits more prominent linear behavior, reaching a $R^{2}=0.620$. SVR, on its part, obtains its best results when the database is reduced with non-linear PCA and feature selection, reducing the effect of dimensionality. Thus, it becomes apparent that the attributes of both linear and SVC are not sufficient to deal with this dimensionality problem, and extra dimensionality reduction and feature selection techniques are necessary to be included in their analytical pipelines, even though their performance results are worse than those of RF. For Random Forest, even focusing on the RAW dataset, it is observed that for Mn (kg·mol)^−1^, it achieves a value of 0.777, whereas the best values are attained by employing a simple PCA with explained variance and RF as the feature selector. Similarly, in the case of $\frac{Mw}{Mn}$, where RAW yields a total of 0.834, there is an approximately 4% improvement to 0.861 using PCA with an rbf kernel and explained variance alongside RF as a feature selector. A slightly higher gain is observed in the conversion rate, where the value increases from 0.886 to 0.937 with PCA using an rbf kernel and explained variance, plus XGBoost as the feature selector. From this, it can be inferred that the characteristics of RF are most suitable for resolving the problem for all selected target magnitudes.

On the other hand, when examining the best solutions, some important conclusions emerge. First, all three top solutions (highlighted in red) use dimensionality reduction with explained variance as the method for selecting the number of features. This clearly underscores the important of dealing with the dimensionality problem when more descriptors than samples are included in the database. Also, another key aspect to be highlighted is the fact that all the best solutions implement feature selection, using RF or XGB indistinctly, which means that boosting methodologies do not improve bagging ones, making the models in the ensemble equally important.

**Figure 7:**
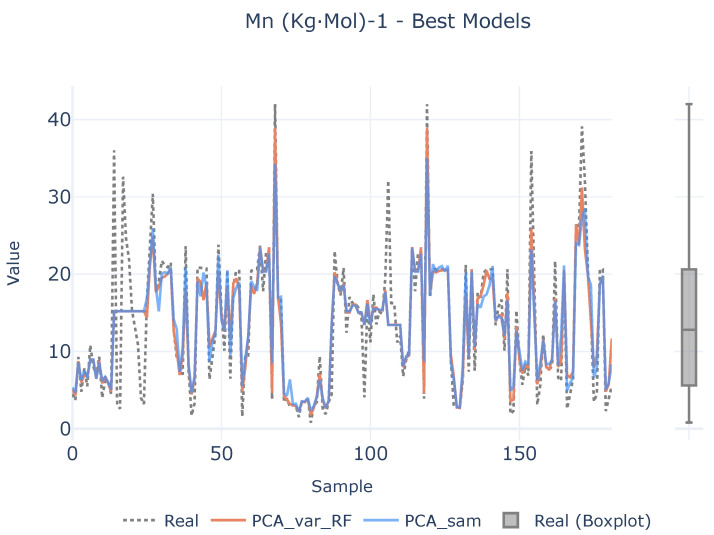
Best models for Mn (kg·mol)^−1^. The left plot illustrates the prediction adjustment from the top two models alongside the actual values, while the right plot displays a boxplot of real Mn (kg·mol)^−1^ values.

**Figure 8:**
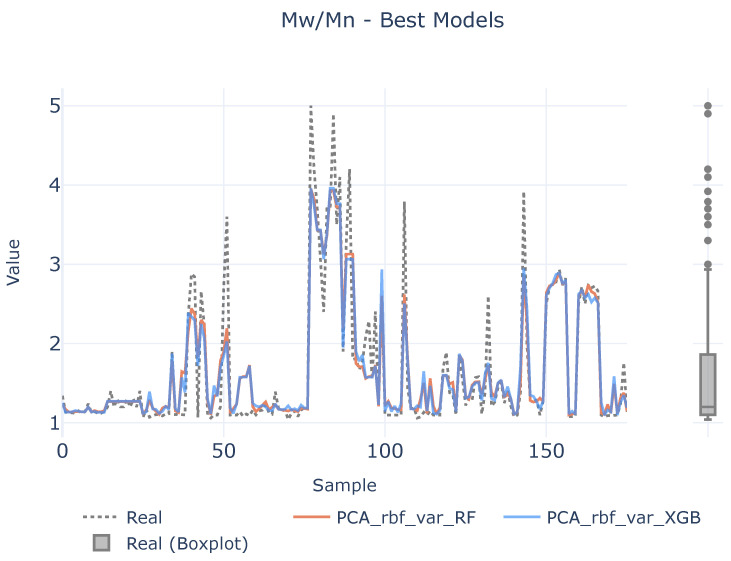
Best models for $\frac{Mw}{Mn}$. The left plot illustrates the prediction adjustment from the top two models alongside the actual values, while the right plot displays a boxplot of the real $\frac{Mw}{Mn}$ values.

**Figure 9:**
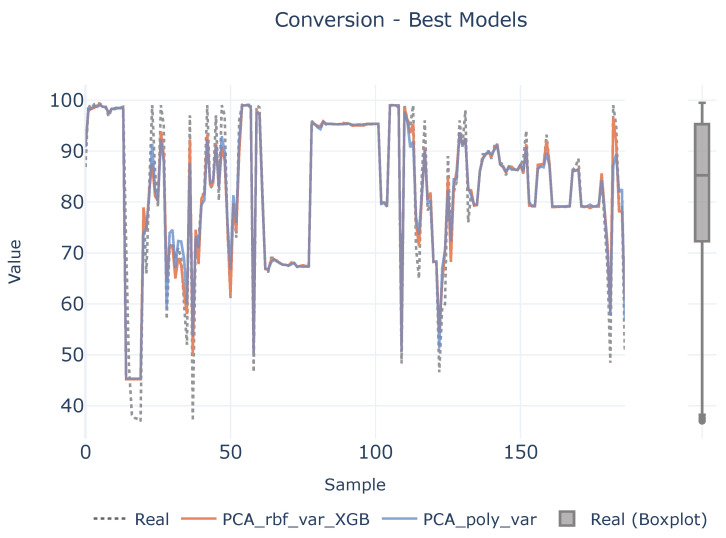
Best models for conversion. The left plot illustrates the prediction adjustment from the top two models alongside the actual values, while the right plot displays a boxplot of the real conversion values.

In order to see if there is a lack of data to infer the causal relationships between descriptors and target variables, a benchmark between the best two defined analytical pipelines and real values are shown in Figure 7, Figure 8 and Figure 9 for each target magnitude, respectively. On the right of each graph, there is a boxplot representing a graphical representation of the statistical distribution of each target magnitude. As it is clearly evidenced in these figures, the analytical pipelines perform worse in areas where outliers are present or they are close to the wings of their statistical distributions as depicted in Figure 8. On their counterparts, the provided results are better where the density of the statistical distributions is higher.

These novel results open the possibility of introducing experimental design techniques to deal with the mapping of the target’s probability distributions in the desired manner (uniformly in the solution space) and thus suggest to the experts new experiments that continue to enrich the newly created database. Moreover, this process can be applied automatically to new samples.

## 5. Conclusions

Through this study, the challenges and uncertainties inherent in the synthesis of polymers through the copolymerization of epoxides with CO_2_ have been addressed. The work has focused on leveraging Artificial Intelligence to mitigate the trial-and-error nature of polymer design, thereby reducing the costs associated with unsuccessful experimentation. Moreover, the first database correlating the structure of the epoxy comonomer, the catalyst employed, and the experimental conditions of polymerization to the reaction’s success has been proposed. This database provides a valuable resource for researchers in materials science, offering insights into the factors influencing polymerization outcomes. Three different targets magnitudes have been studied, the molecular weight (Mn (kg·mol)^−1^), polydispersity index ($\frac{Mw}{Mn}$), and conversion rate.. An analytical pipeline has been defined that allows the database to be exploited automatically and incrementally as new samples join. The proposed analytical pipeline, based on ML techniques, automatically finds the best data preprocessing schema and the models to employ to effectively exploit the constructed database, addressing the dimensionality problem and delivering promising results. Looking ahead, expansion of the database will enable the development of more sophisticated AI techniques. The presented results show that from the earliest stage of experimentation, Artificial Intelligence could provide useful information for anticipating the success of the experimentation.

We are aware that, compared to the datasets employed in other knowledge areas where Machine Learning is commonly used, the described database is not excessively large. This is because, although this polymerization is not a new reaction, the data in the literature are scarce and not homogeneous, a typical problem in the polymer field. In this sense, this work can be considered the starting point of an iterative work since the dataset can be constantly fed with upcoming results, enabling the development of more sophisticated ML techniques. Moreover, looking ahead, other experimental parameters such as the concentration of starting materials and the reaction scale could be included in the database in order to study their influence on the success of the polymerization.

The results obtained could reduce the time required for the polymer design process, which may not only contribute to the reduction in atmospheric CO_2_ and lead to more sustainable polymer lifecycles but also enhance the design capabilities of new materials. This could have a direct impact on a wide range of areas, including agriculture, medicine, and electronics.

## Figures and Tables

**Table 1:** Grid Search optimization: Variables enclosed in brackets denote a discrete list of values for target variable, while variables within parentheses denote a range, defined by their start, end, and step values.

SVR	
Name	Possible Values
kernel	[poly, rbf]
C	(0.1, 1.0, 0.3)
epsilon	(0.0, 1.0, 0.1)
RF	
Name	Possible Values
max_depth	(1, 20, 2)
min_samples_split	(2, 20, 2)
min_samples_leaf	(1, 20, 2)
max_features	[sqrt, log2, None] + (0.1, 0.9, 0.1)
n_estimators	[10, 50, 100, 150, 200]

**Table 2:** Results for each target variable and model trained at different data preprocessing stages. In bold are the five best solutions for each data combination. Highlighted in red is the best solution, and highlighted in blue is the second best solution.

	*Mn* (kg·mol)^−1^			*Mw/Mn*			Conversion		
	**LR**	**SVR**	**RF**	**LR**	**SVR**	**RF**	**LR**	**SVR**	**RF**
RAW	−72.130	0.102	0.777	$-3.93\times 10^{4}$	−0.317	0.834	$-3.67\times 10^{3}$	0.292	0.886
PCA_poly_sam	$-3.76\times 10^{12}$	0.095	0.750	$-2.94\times 10^{10}$	0.200	0.793	$-5.55\times 10^{10}$	0.146	**0.909**
PCA_poly_sam_RF	0.095	0.152	0.441	0.068	0.385	0.723	0.270	0.137	0.768
PCA_poly_sam_XGB	0.016	0.049	0.224	0.070	0.221	0.743	0.112	0.349	0.829
PCA_poly_var	$-3.76\times 10^{12}$	0.095	0.719	$-2.94\times 10^{10}$	0.200	0.845	$-5.55\times 10^{10}$	0.146	**0.930**
PCA_poly_var_RF	0.095	0.152	0.444	0.076	0.393	0.709	0.178	0.113	0.716
PCA_poly_var_XGB	0.016	0.049	0.209	0.070	0.221	0.751	0.112	0.349	0.829
PCA_rbf_sam	$-2.83\times 10^{10}$	0.289	0.718	$-1.64\times 10^{12}$	0.704	0.834	$-7.44\times 10^{6}$	0.404	0.901
PCA_rbf_sam_RF	0.197	0.221	0.537	0.456	0.604	0.816	0.620	0.387	0.866
PCA_rbf_sam_XGB	0.003	0.005	0.424	0.192	0.548	**0.846**	0.417	0.385	0.865
PCA_rbf_var	$-2.83\times 10^{10}$	0.289	0.778	$-1.64\times 10^{12}$	0.704	0.843	$-7.44\times 10^{6}$	0.404	0.849
PCA_rbf_var_RF	0.198	0.222	0.556	0.459	0.601	**0.861**	0.581	0.385	0.868
PCA_rbf_var_XGB	0.003	0.005	0.481	0.192	0.548	**0.850**	0.417	0.385	**0.937**
PCA_sam	$-3.87\times 10^{22}$	0.224	**0.782**	$-2.42\times 10^{20}$	0.461	0.779	$-4.00\times 10^{23}$	0.305	0.891
PCA_sam_RF	0.067	0.239	0.639	0.183	0.411	0.750	0.492	0.308	0.875
PCA_sam_XGB	0.067	0.239	0.594	0.156	0.373	0.725	0.305	0.394	0.899
PCA_var	0.208	0.211	**0.784**	0.437	0.391	0.824	0.608	0.308	0.844
PCA_var_RF	0.183	0.226	**0.794**	0.253	0.422	0.740	0.573	0.317	0.848
PCA_var_XGB	0.105	0.187	0.711	0.437	0.391	0.793	0.608	0.308	0.859
RF	0.266	0.103	0.605	0.470	0.451	0.777	0.213	0.398	0.744
XGB	0.191	0.186	0.191	0.150	0.234	0.730	0.235	0.316	0.768

## Data Availability

Finally, this section includes a link to the paper’s repository. It contains the initial database and all the code developed in this research to ensure transparency and clarity. Additionally, the source code for the new methods is provided via a publicly accessible service and distributed under an open-source license. https://git.code.tecnalia.com/aritz.martinez/co2-polycarbonates (accessed on 16 October 2024).
